# Analysis of Gender-Dependent Personal Protective Behaviors in a National Sample: Polish Adolescents’ COVID-19 Experience (PLACE-19) Study

**DOI:** 10.3390/ijerph17165770

**Published:** 2020-08-10

**Authors:** Dominika Guzek, Dominika Skolmowska, Dominika Głąbska

**Affiliations:** 1 Department of Food Market and Consumer Research, Institute of Human Nutrition Sciences, Warsaw University of Life Sciences (SGGW-WULS), 02-776 Warsaw, Poland; 2 Department of Dietetics, Institute of Human Nutrition Sciences, Warsaw University of Life Sciences (SGGW-WULS), 02-776 Warsaw, Poland; dominika_skolmowska@sggw.edu.pl (D.S.); dominika_glabska@sggw.edu.pl (D.G.)

**Keywords:** Coronavirus-19, COVID-19, SARS-CoV-2, hand hygiene, personal protective behaviors, adolescents, national population-based study, PLACE-19 Study

## Abstract

During the coronavirus-19 disease (COVID-19) pandemic, the basic strategy that is recommended to reduce the spread of the disease is to practice proper hand hygiene and personal protective behaviors, but among adolescents, low adherence is common. The present study aimed to assess the gender-dependent hand hygiene and personal protective behaviors in a national sample of Polish adolescents. The Polish Adolescents’ COVID-19 Experience (PLACE-19) Study was conducted in a group of 2323 secondary school students (814 males, 1509 females). Schools were chosen based on the random quota sampling procedure. The participants were surveyed to assess their knowledge and beliefs associated with hand hygiene and personal protection, as well as their actual behaviors during the COVID-19 pandemic. The majority of respondents gave proper answers when asked about their knowledge. However, females displayed a higher level of knowledge (*p* < 0.05). Most of the respondents declared not leaving home, handwashing, using alcohol-based hand rub, avoiding contact with those who may be sick, and avoiding public places as their personal protective behaviors. They declared using face masks and gloves after the legal regulation requiring people to cover their nose and mouth in public places was enacted in Poland. Regarding the use of face masks and not touching the face, no gender-dependent differences were observed, while for all the other behaviors, females declared more adherence than males (*p* < 0.05). Females also declared a higher daily frequency of handwashing (*p* < 0.0001) and washing their hands always when necessary more often than males (68.2% vs. 54.1%; *p* < 0.0001). Males more often indicated various reasons for not handwashing, including that there is no need to do it, they do not feel like doing it, they have no time to do it, or they forget about it (*p* < 0.0001), while females pointed out side effects (e.g., skin problems) as the reason (*p* = 0.0278). Females more often declared handwashing in circumstances associated with socializing, being exposed to contact with other people and health (*p* < 0.05), and declared always including the recommended steps in their handwashing procedure (*p* < 0.05). The results showed that female secondary school students exhibited a higher level of knowledge on hand hygiene and personal protection, as well as better behaviors, compared to males. However, irrespective of gender, some false beliefs and improper behaviors were observed, which suggests that education is necessary, especially in the period of the COVID-19 pandemic.

## 1. Introduction

Due to the outbreak of COVID-19 (coronavirus-19 disease) caused by SARS-CoV-2 (severe acute respiratory syndrome coronavirus 2), the World Health Organization (WHO) announced a global pandemic alert [1] and developed a “strategic preparedness and response plan” to reduce the disease transmission [2]. The complex strategy recommends the practice of proper hand hygiene, including either washing with soap and water or rubbing with an alcohol-based hand rub [3], as well as the use of personal protective equipment, including gloves, medical/surgical face masks, or face shields [4]. The other important recommendations are to practice isolation for people who are ill or infected, quarantine for those who are suspected as exposed [5], and social distancing for all the others [6]. The role of hand hygiene [7,8,9] and other personal protective behaviors [10,11,12] in the prevention of COVID-19 is also emphasized by major international authorities, as for the time being, there is no vaccine developed for the disease [13] and so prevention is the only way to reduce the transmission of SARS-CoV-2.

It should be considered that the governments have the responsibility to take accurate actions and implement adequate measures to evoke behavioral changes among people in order to fight the pandemic. One such important action is providing proper information about personal protective behaviors for illness prevention because uncertainty about personal protection could lead to anxiety, depression, and distress [14]. All these will only result in panic, rather than effective behavioral changes for reducing SARS-CoV-2 transmission [15]. In this regard, the governments must prioritize changing personal behaviors into those that might fight against the COVID-19 pandemic through proper education [16]. This is crucial, especially considering the fact that some studies have indicated low trust in governments due to their inefficient actions during the COVID-19 outbreak [17].

In Poland, the first COVID-19 case was confirmed on 4 March 2020. Over 24 days after the confirmation of the first case, the total number of confirmed cases rose to 1389, with 34,000 laboratory tests being performed and 16 deaths due to the disease (the average age of the infected people was 65.5 years, and 81.2% of them were males) [17]. Among the cases confirmed until 30 April 2020, 55.7% were females. The mean age of the patients was 50.6 years, with the youngest confirmed case being less than 1 year old and the oldest being 103 years old [18].

Reducing the risk of exposure to SARS-CoV-2 is crucial for the high-risk groups, including the immunocompromised and elderly individuals [19], while young people are those who may be responsible for the spread of the disease, as they are more frequently engaged in social activities [20]. Hence, the WHO recommended that the adolescents should strictly follow the guidelines to prevent disease transmission, even if they believe that they are unlikely to get ill [21]. Similarly, the United Nations International Children’s Emergency Fund (UNICEF) emphasized that adolescents and youth can also possibly contract and transmit SARS-CoV-2, and so they should be well informed, resourced, and educated about COVID-19 and its preventive measures [22]. In addition, the recent Cochrane review by Nussbaumer-Streit et al. [23] highlighted the role of this population group in the spread of COVID-19 and concluded that closing schools, following social distancing, and implementing other public health measures can increase the effectiveness of quarantine applied to improve disease control. Therefore, hand hygiene and personal protective behaviors are indicated for adolescents as essential [24] to significantly reduce the risk of COVID-19 transmission [25]. However, adherence seems to be challenging for this age group, as the recent study of Chen et al. [26] conducted among school students in Wuhan (China) indicated that in February 2020, only 42% and 52% of them followed good handwashing and mask-wearing behaviors, respectively. These percentages are very low considering the fact that student status was associated with a greater psychological impact due to the pandemic and higher levels of stress, anxiety, and depression compared to the other population groups in China in the analyzed period [27].

However, not only young age or student status can be considered as determinants of low adherence to the recommended hand hygiene and other personal protective behaviors; there are other factors, such as gender, that also influence the level of adherence. Gender is commonly indicated as a significant determinant of hand hygiene behaviors. The study of Suen et al. [28] revealed that the knowledge of general hand hygiene was relatively poor in a Hong Kong population, but female respondents displayed a higher level of knowledge than males. Similarly, it was shown that women working in the critical care unit washed their hands after contact with patients more often than the male workers [29]. Furthermore, in the study of Anderson et al. [30], a higher share of female students declared washing their hands before exiting the restroom, compared to the male students. In the study of Mariwah et al. [31], a higher proportion of Ghanian women declared washing their hands before leaving the toilet, while a higher share of women washed both their hands, in comparison to men. However, such observations were not reported by some authors. For instance, no significant differences in hand hygiene behaviors were observed between female and male respondents by Park et al. [32] in their study on physicians, by van de Mortel et al. [29] in their study on other healthcare workers, and by Sultana et al. [33] in their study on university students.

The recent study by Zhong et al. [34], conducted in Hubei province in January 2020, indicated that gender was a determinant of mask-wearing and social isolation behaviors during the COVID-19 pandemic. This study is the only one that was performed specifically in the period of this pandemic, and hence, it is not clear if such an influence exists in other countries as well. However, a meta-analysis published regarding the association between gender and protective behaviors in response to respiratory epidemics and pandemics (avian influenza, swine influenza, Middle East respiratory syndrome (MERS), and severe acute respiratory syndrome (SARS)) indicated that women are much more likely to practice behaviors such as using face masks, handwashing, and avoiding public transport than men to reduce the spread of infectious diseases [35]. Taking into consideration that only a limited number of studies have focused on the hand hygiene and personal protective behaviors of people during epidemiological states, including only one study [34] in the case of COVID-19, and that they were conducted only among specific populations, there is a need to conduct such studies among different populations in order to confirm if this trend is common or varies depending on the situation.

Therefore, it must be emphasized that while gender was found to be an important determinant of compliance with hand hygiene behaviors before the period of the COVID-19 pandemic, its current influence on the hand hygiene and personal protective behaviors remains unknown. Taking this into account, the present study aimed to assess and compare the hand hygiene and personal protective behaviors between male and female respondents in a national sample of Polish adolescents within the Polish Adolescents’ COVID-19 Experience (PLACE-19) Study population.

## 2. Materials and Methods

### 2.1. Studied Population

The PLACE-19 Study was conducted in Poland on a national sample of Polish secondary school students and analyzed their hand hygiene and personal protective behaviors. The schools were chosen based on the random quota sampling procedure conducted for each region of Poland (within voivodeships and counties) in cooperation with the local boards of education. According to the sampling procedure, secondary school students aged 15–20 years were invited to participate. In Poland, secondary school education is not compulsory, but the vast majority of adolescents are taking it up, as education is compulsory until the age of 18 years [36]. Thus, for the secondary schools in Poland, the net enrollment rate (NER) is estimated as 89.38% [37].

The present study was carried out at the Institute of Human Nutrition Sciences, Warsaw University of Life Sciences (WULS-SGGW). It was conducted in accordance with the Declaration of Helsinki, and all the procedures associated with sampling and data gathering were approved by the Ethics Committee of the Institute of Human Nutrition Sciences (WULS-SGGW).

Poland is divided into six main geographic regions with 16 administrative units, called voivodeships. The shape and size of these voivodeships depend on historic, cultural, economic, and geographic factors, and each voivodeship is further divided into counties [38]. To obtain a proportional sample of students from all the regions of Poland, the schools were invited to participate in the study based on their geographical distribution within voivodeships and counties. The random quota sampling procedure was applied and two stages of stratified sampling were carried out (Table 1), as described in the previous study [39], to obtain the sample from each segment based on administrative units of Poland equally distributed within voivodeships/regions. It was based on random sampling of counties for voivodeships and random sampling of schools for counties.

The principal of each school chosen for the study was informed about the study purpose and that participation of their school in the study was voluntary and would be arranged by the local boards of education if necessary. If agreed, the principal forwarded an electronic link for the questionnaire to the students who expressed their will to participate voluntarily in the study. The applied questionnaire did not collect any personal or sensitive data, and all the answers were anonymous. If needed, after 1 week of the first stage and after 1 week of the second stage, the principal was sent a reminder about the study.

The inclusion criteria to be fulfilled by the students for participation in the study were as follows: a student of a chosen school, aged 15–20 years, who provide informed consent to participate. The exclusion criteria were as follows: lack of any data and presence of unreliable answers in the questionnaire.

**Figure 1:**
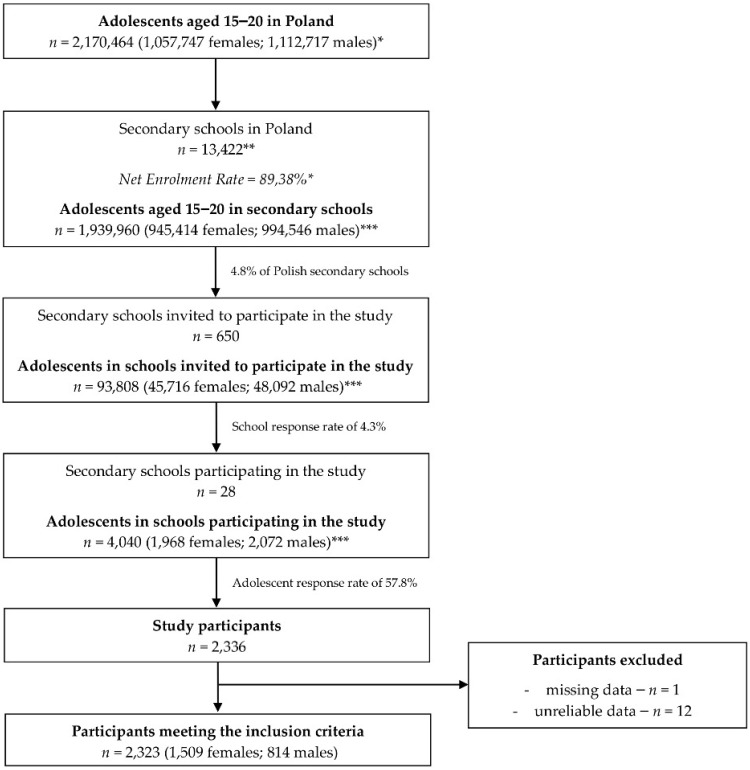
Scheme of recruitment of the studied Polish adolescents. * Data from the Central Statistical Office (CSO) in Poland [37,40]; ** data from the Polish Ministry of National Education [41]; *** estimated based on the data from CSO.

Based on the described procedure, a total of 2323 secondary school students (814 males and 1509 females) were included in the study and provided complete and reliable questionnaires. The scheme of recruitment of the participants is presented in Figure 1.

### 2.2. Data Collection

After the first COVID-19 case was confirmed in Poland on 4 March 2020 [17] and the WHO declared the disease as a global pandemic on 11 March 2020 [1], both primary and secondary school education was suspended in the country as of 12 March 2020 [42]. During this period, Polish secondary schools had to adopt a remote education system, as all the citizens were recommended to reduce personal contact, not leave their households unless necessary, and include hygiene behaviors into their daily routine. The study was conducted in this period of remote education, but on 16 April 2020, an additional precept was announced that all the citizens should wear a face mask when leaving their households, as having the nose and mouth covered in public places was obligatory [43].

The study used an electronic questionnaire that had questions about hand hygiene and personal protective behaviors, as well as questions about gender, age, and name of the school (to verify the inclusion criteria). No personal or sensitive data or information that would allow the identification of respondents were collected.

In the questions on hand hygiene and personal protective behaviors, respondents were asked about their knowledge and beliefs, as well as about their actual behaviors. Such an approach in the case of hand hygiene and personal protective behaviors is also applied by other authors, and they combine issues of knowledge/beliefs and actual behaviors in a common questionnaire [44,45,46]. Combining questions about knowledge/beliefs and actual behaviors allows assessing if improper behaviors are accompanied by lack of knowledge or any false beliefs.

The questions about knowledge and beliefs (Supplementary Table S1) asked the respondents’ which choice of method in their opinion is better for proper hand hygiene and personal protection. They were presented five pairs of behaviors in everyday situations, from which they had to choose one as the better option (ensuring better protection) or indicate that they are equally good. Alternatively, they could choose to not provide an answer if they did not know which one was better (close-ended question). The pairs of behaviors were presented as follows: (1) not leaving home/using a face mask; (2) handwashing/using gloves; (3) using soap/using alcohol-based hand rub; (4) using liquid soap/using a soap bar; and (5) using paper towels/using a hand dryer. The questions that were asked to assess knowledge and beliefs were based on the questions from the Hand Hygiene Knowledge Questionnaire for Health-Care Workers developed by the WHO [47]—the referred questionnaire presents the same format of questions which are formulated to compare two different methods of hand hygiene. However, the questions asked in the present study were developed to be suitable for students and were specific for the period of COVID-19 pandemic, as there is no dedicated questionnaire available for such situation. When analyzing the respondents’ answers, it was interpreted that in everyday situations, not leaving home is better (ensures better protection) than using a face mask [48], handwashing is better than using gloves [49], using soap is better than using an alcohol-based hand rub [49], using liquid soap is better than using a soap bar [50], and using paper towels is better than using a hand dryer [51], as in Poland the vast majority of hand dryers do not have a UV light.

The additional question associated with knowledge and beliefs was about the required time of handwashing according to their opinion. It was formulated as a close-ended question with the following options: less than 5 s; 5–10 s; 11–20 s; 21–40 s; more than 40 s; time does not matter; and do not know which one is a proper answer. The question and the time categories were proposed by Park et al. [44]. When analyzing the responses, it was interpreted that handwashing for more than 20 s is required [8,9,52], but the WHO recommends a duration of more than 40 s [53], especially in the context of COVID-19 [54], so an additional answer of more than 40 s was included in the answers for the original question of Park et al. [44]. The answers 21–40 s and more than 40 s were treated as appropriate.

The questions regarding actual hand hygiene and personal protective behaviors (Supplementary Table S2) that are currently practiced during the COVID-19 pandemic were asked, so the respondents were asked to consider the period of this pandemic, which for them was defined as the period of remote education (which is easier for the students to recognize than the COVID-19 pandemic officially declared by the WHO).

The respondents were asked about their personal protective behaviors and those of their vulnerable relatives (e.g., elderly ones in their families). The behaviors that were asked are as follows: not leaving home; using a face mask; not touching the face; using gloves; handwashing; using alcohol-based hand rub; avoiding contact with those who may be sick; avoiding public places; taking medications or dietary supplements; and other (close-ended multiple-choice question). These indicated personal protective behaviors were chosen based on a previous focus group interview conducted to define behaviors applied either by the participants of the group, or by other people due to the COVID-10 pandemic to reduce the risk of infection, and were specified by the participants as either not applied previously or applied more often during the COVID-19 pandemic than before. When analyzing the answers for this question, the respondents were divided into two subgroups (surveyed before and after 16 April 2020) due to the fact that as of 16 April 2020, having the nose and mouth covered in public places was obligatory [43].

The respondents were asked about their hand hygiene behaviors including the following: the frequency of handwashing, reasons for not handwashing, circumstances of handwashing, and procedure applied.

To specify the frequency of handwashing, the respondents were asked to choose one of the following options: not washing at all; 1–2 times a day; 3–5 times a day; 6–10 times a day; 11–15 times a day; 16–20 times a day; 21–30 times a day; and more than 30 times a day (close-ended question). The question was formulated based on the study by Merk et al. [55], but different options were applied due to the fact that during the COVID-19 pandemic, higher frequencies were expected than usual.

To specify the reasons for not handwashing, the respondents were asked to choose all the relevant answers from the following: in my opinion, there is no need to do it; I don’t feel like doing it; I have no time to do it; I am forgetting about it; it is constricted (e.g., there is no soap, or no bathroom nearby); due to side effects (e.g., skin problems due to frequent contact with soap); other; and not applicable—if they always wash their hands (close-ended multiple-choice question). These indicated reasons for not handwashing were chosen based on a previous focus group interview conducted to identify the reasons among the group participants.

To specify the circumstances of handwashing, the respondents were asked questions based on the Handwashing Habits Questionnaire (HHQ), developed by Tüzün et al. [56] on the basis of the questionnaire by Üner et al. [57], which is commonly applied [33,58,59,60]. HHQ is a tool used to verify the handwashing habits in various circumstances that are in agreement with the recommendations of the WHO [7,53], the UNICEF [8], and the Centers for Disease Control and Prevention (CDC) [9,52,61]. Each of the listed circumstances of handwashing was to be assigned to handwashing never, sometimes, or always. The circumstances that were included in the analysis were directly associated with the COVID-19 protective behaviors, namely socializing, being exposed to contact with other people, and health, and were listed as follows: after coming back home; after handshaking; after using public transportation; after money exchange; before touching sick people; after touching sick people; after nose blowing; after sneezing; and after coughing.

To specify the procedure applied for handwashing, the respondents were asked questions about each step of the procedure to be assigned as included in their procedure never, sometimes, or always. The steps asked were as follows: folding sleeves (while it was also possible to declare as not applicable), removing watches and bracelets (while it was also possible to declare as not applicable), removing rings before or during handwashing (while it was also possible to declare as not applicable), using soap, using warm water, soaking hands before using soap (while it was also possible to declare as not applicable if they do not use soap), spreading soap lather throughout the hands (while it was also possible to declare as not applicable if they do not use soap), turning the faucet off with hand, and drying hands with a towel. The question and the steps included are commonly used in a number of studies dealing with handwashing procedure [58,62,63,64]. For the majority of steps, including them in the applied procedure was interpreted as needed according to the handwashing guidelines [7,8,9,52], but for the step described as “turning the faucet off with hand”, including it was interpreted as incorrect due to the fact the faucet should not be touched barehanded after handwashing.

### 2.3. Statistical Analysis

The sample size was calculated for the population of Polish adolescents (2,170,464, based on the data from the Central Statistical Office (CSO) in Poland [40]), at the confidence level of 95% and margin of error of 5%, while a percentage of 50% was assumed and the required sample size was estimated as 384 respondents. Thus, the gathered sample of 2323 respondents was interpreted as sufficient.

The answers provided by the male and female respondents were compared using the chi^2^ test. *p* ≤ 0.05 was interpreted as a significant difference between groups. Statistical analysis was conducted using Statgraphics Plus for Windows 4.0 (Statgraphics Technologies Inc., The Plains, VA, USA).

## 3. Results

### 3.1. Knowledge and Beliefs Associated with Hand Hygiene and Personal Protective Behaviors

The knowledge and beliefs of the questioned sample of Polish secondary school students interpreted based on the choice of the method they believed is better for proper hand hygiene and personal protection are described in Table 2. For not leaving home vs. using a face mask, the majority of respondents indicated the appropriate answer and no significant gender-dependent differences were noted (*p* = 0.0521). However, for the other behaviors, differences were observed. For questions on handwashing vs. using gloves, and using soap vs. using alcohol-based hand rub, the majority of respondents were not able to choose the proper answer, and regardless of gender, many indicated that those behaviors are equally good. Handwashing was chosen as proper behavior compared to using gloves by many male respondents (*p* < 0.0001), while using soap was chosen as appropriate compared to using alcohol-based hand rub mainly by females (*p* < 0.0001). For using liquid soap vs. using a soap bar, and using paper towels vs. using a hand dryer, the majority of females chose proper behaviors, while the number of male respondents who indicated proper answers was lower (*p* = 0.0001 and *p* = 0.0441, respectively).

The knowledge and beliefs of the questioned sample of Polish secondary school students interpreted based on the time of handwashing they believed as required are described in Table 3. The majority of respondents indicated proper answers, but a significant gender-dependent difference was observed, as a higher share of female respondents chose the proper answers compared to males (*p* < 0.0001).

### 3.2. Actual Hand Hygiene and Personal Protective Behaviors

The personal protective behaviors declared by the questioned sample of Polish secondary school students, before and after the implementation of the legal regulation that people should cover their nose and mouth in public places, are presented in Table 4. During both the periods, the majority of respondents declared not leaving home, handwashing, using alcohol-based hand rub, avoiding contact with those who may be sick, and avoiding public places, which are the recommended personal protective behaviors. However, after the enactment of the legal regulation that the nose and mouth should be covered in public places, the majority of respondents declared that they were not only using a face mask but also using gloves, which were not commonly indicated in the previous period. When comparing male and female respondents, it was observed that there were no gender-dependent differences in the case of using a face mask and not touching the face (both associated with an in-force obligation) in the period after the legal regulation was enacted, while for all the other behaviors, females declared practicing them more often than males (*p* < 0.05).

The personal protective behaviors of vulnerable relatives declared by the questioned sample of Polish secondary school students, before and after the implementation of the legal regulation that people should cover their nose and mouth in public places, are presented in Table 5. During both the periods, the majority of respondents declared that their vulnerable relatives were not leaving home, handwashing, using alcohol-based hand rub, avoiding contact with those who may be sick, and avoiding public places (similarly as they declared for themselves), which are the recommended personal protective behaviors. However, after the enactment of the legal regulation that the nose and mouth should be covered in public places (16 April 2020), the majority of respondents declared that they were not only using a face mask but also using gloves (similarly as they declared for themselves), which were not commonly indicated in the previous period. When comparing male and female respondents, some gender-dependent differences were observed, as female respondents declared more often than male respondents that their vulnerable relatives were not leaving home (*p* = 0.0214 for the period after 16 April 2020), using a face mask (*p* = 0.0025 for the period before 16 April 2020), using gloves (*p* < 0.0001 and *p* = 0.0003 for the period before and after 16 April 2020, respectively), handwashing (*p* = 0.0408 for the period after 16 April 2020), using alcohol-based hand rub (*p* = 0.0016 for the period before 16 April 2020), avoiding contact with those who may be sick (*p* = 0004 and *p* = 0.0113 for the period before and after 16 April 2020, respectively), and taking medications or dietary supplements (*p* = 0.0020 and *p* = 0.0003 for the period before and after 16 April 2020, respectively). On the other hand, male respondents declared more often than female respondents that their vulnerable relatives were avoiding public places (*p* = 0.0257 for the period before 16 April 2020).

The frequency of handwashing declared by the questioned sample of Polish secondary school students is presented in Table 6. When comparing male and female respondents, it was observed that females declared a higher daily frequency of handwashing than males (*p* < 0.0001). At the same time, it may be indicated that the COVID-19 pandemic influenced the frequency of handwashing for both males and females (Supplementary Table S3).

The reasons for not handwashing declared by the questioned sample of Polish secondary school students are presented in Table 7. When comparing male and female respondents, females more often declared washing their hands always when necessary (68.2% vs. 54.1%; *p* < 0.0001), while males indicated various reasons for not handwashing, including that in their opinion there is no need to do it (*p* < 0.0001), they don’t feel like doing it (*p* < 0.0001), they have no time to do it (*p* < 0.0001), or they are forgetting about it (*p* < 0.0001). On the other hand, compared to males, females more often indicated that they were not handwashing due to side effects (e.g., skin problems due to frequent contact with soap) (*p* = 0.0278). At the same time, it may be indicated that the COVID-19 pandemic influenced reasons for not handwashing for both males and females (Supplementary Table S4).

The circumstances of handwashing associated with socializing and being exposed to contact with other people as declared by the questioned sample of Polish secondary school students, based on the Handwashing Habits Questionnaire [56], are presented in Table 8. When comparing male and female respondents, it was observed that females more often declared washing their hands always after coming back home (*p* < 0.0001), after handshaking (*p* = 0.0041), after using public transportation (*p* < 0.0001), and after money exchange (*p* < 0.0001). At the same time, the majority of questioned students declared handwashing always after coming back home, after using public transportation, and in the case of females, after money exchange. At the same time, it may be indicated that the COVID-19 pandemic influenced circumstances of handwashing associated with socializing and being exposed to contact with other people for both males and females (Supplementary Table S5).

The circumstances of handwashing associated with health, as declared by the questioned sample of Polish secondary school students, based on the Handwashing Habits Questionnaire [56], are presented in Table 9. When comparing male and female respondents, females more often declared washing their hands always after touching sick people (*p* = 0.0168), after nose blowing (*p* = 0.0004), after sneezing (*p* = 0.0006), and after coughing (*p* = 0.0069). At the same time, the majority of participants declared washing their hands always after touching sick people. At the same time, it may be indicated that the COVID-19 pandemic influenced circumstances of handwashing, associated with health for both males and females (Supplementary Table S6).

The procedure of handwashing declared by the questioned sample of Polish secondary school students is presented in Table 10. When comparing male and female respondents, females more often declared always including the following steps in their procedure of handwashing: folding sleeves (*p* = 0.0045), removing watches and bracelets (*p* = 0.0053), removing rings before or during handwashing (*p* = 0.0076), using soap (*p* = 0.0185), using warm water (*p* = 0.0001), soaking hands before using soap (*p* = 0.0064), and spreading soap lather throughout the hands (*p* = 0.0163). On the other hand, compared to females, males more often declared turning the faucet off with hands (*p* = 0.0004), which is incorrect, and drying hands with a towel (*p* = 0.0120). At the same time, the majority of questioned students declared always including the following steps when handwashing: using soap, using warm water, soaking hands before using soap, spreading soap lather throughout the hands, drying hands with a towel, and in the case of females, folding sleeves. At the same time, it may be indicated that the COVID-19 pandemic influenced the procedure of handwashing for both males and females (Supplementary Table S7).

## 4. Discussion

The current COVID-19 pandemic is a major healthcare crisis for the global population [65]. In the case of children and adolescents, the COVID-19 situation seems to have a prominent influence on their lifestyle behaviors [66]. However, a low compliance with the recommended protective measures is observed in this group, which is emphasized as an important problem for the entire population as reducing the disease transmission is considered as the main target [67]. Insufficient adherence is observed among adolescents in spite of the fact that practicing proper hand hygiene and other personal protective behaviors is the only way to limit the risk of SARS-CoV-2 infection in the general population [68].

Certain sociodemographic factors, such as the level of education, habitual residence, and gender, are associated with compliance with hand hygiene behaviors, among which gender, is the most influential [28]. In a number of studies, it is reported that female respondents display better hand hygiene behaviors than males, independent of the studied group, as women are more likely than men to wash their hands, wash both hands, wash hands properly, and wash hands more often [28,29,30,31]. Similarly, in a systematic review analyzing the influence of gender on the health risk behaviors, it was indicated that handwashing behavior is included in the everyday hygienic behaviors mainly in the case of women [69]. Moreover, a significantly higher share of women than men practice proper hand hygiene behaviors when being observed by others, or in the presence of a sign reminding them to do it [70]. In addition, the applied educational intervention was reported to have a more significant effect on women than men, as in the postintervention period significantly better hand hygiene behaviors were observed among women than in the preintervention period, while in the case of men, no such difference was observed [71]. However, it was also stated that gender and washroom characteristics are related, as women’s washrooms are more likely to be clean than those of men, as well as soap is more likely to be available in women’s washrooms than in men’s washrooms [31].

Similar to the previous studies, in the present one, some important differences were observed between male and female respondents, because for a majority of the assessed hand hygiene and personal protective behaviors, as well as in the case of general knowledge in this area, women fared well and exhibited better behaviors than men. For most of the questions, female respondents gave proper answers and thus showed a higher level of knowledge than males (except for handwashing vs. using gloves, for which more male respondents indicated a more proper answer than females). In the case of using a face mask and not touching the face, no gender-dependent differences were observed, while for all the other assessed behaviors, females declared practicing them more often than males. In addition, females declared a higher daily frequency of handwashing than males and declared washing their hands more often always when necessary, while males indicated various reasons for not handwashing, including that in their opinion there is no need to do it, they do not feel like doing it, they have no time to do it, or they forget about it. Furthermore, females more often declared always washing their hands in various circumstances associated with socializing, being exposed to contact with other people, and health, and declared always including the recommended steps in their handwashing procedure.

In the present study, some personal protective behaviors were not applied by the studied group. This was especially visible when comparing the situations before and after enactment of the legal regulation that people should cover their nose and mouth in public places. Before the enactment, the use of face masks in public places was declared only by 25.7% and 34.5% of male and female respondents, respectively, while after the enactment by 85.6% and 88.9%, respectively; therefore, it can be indicated that only the legal regulation must have forced adolescents to follow this recommendation. This is particularly true in the case of male respondents, as in the period before enactment only a significantly lower share of male respondents were following this recommendation compared to females, while after the enactment, the share of males and females using a face mask was comparable. Similarly, the study by Oosterhoff et al. [72], conducted in the USA, reported that over 60% of adolescents declared that their motivation to engage in social distancing was the fact that their state or city was on lockdown and it was a legal issue, but as an important motivation they declared also their social responsibility or the fact that they do not want others to get sick.

This observation confirms the previously indicated difference between male and female respondents, as it seems that women are more likely to apply personal protective behaviors than men. Similarly, in the review of Bish and Michie [73], which identified the determinants of different protective behaviors practiced during swine flu pandemic, it was indicated that compliance to wearing face masks was associated with gender. The same observations were reported by Lee et al. [74] in their study which stated that in all the required situations (including when taking care of family members with fever, when taking care of family members with respiratory infection, when visiting clinics during peak season or a flu pandemic, when visiting hospitals during peak season or a flu pandemic, and when having respiratory symptoms), females declared a higher frequency of using face masks than males.

The present study was conducted during the COVID-19 pandemic, and while there is only limited evidence indicating the influence of gender on personal protective behaviors [34], some studies have been conducted in similar epidemiological situations of threats forcing people to be more focused on necessary protection. For instance, in a study conducted during the global outbreak of SARS in 2003 in Hong Kong, it was stated that female residents were more likely to wear face masks than males, while the population group that was characterized by the highest frequency of practicing this protective behavior was married women aged 50–59 [75]. Similarly, in a study conducted during the influenza A/H1N1 (human swine flu) outbreak in 2009 in Hong Kong, it was stated that female residents were more likely to wear face masks regularly than males, while the population group that was characterized by the highest frequency of practicing this protective behavior was married 50–60-year old women who were not employed full-time [76]. In the same study, it was also indicated that women and participants with a higher level of education were more likely to wear face masks than males and those with a lower level of education when having the symptoms of an influenza-like illness (ILI) [76]. In the study by Lau et al. [76] that analyzed if respondents were handwashing at least 10 times a day, it was reported that this behavior was mainly followed by married women aged 30–39, while in the case of situation of ILI symptoms, it was practiced by women and participants with a higher level of education. In the study on the influenza A/H1N1 outbreak in Korea, the frequency of handwashing was higher among women than men, especially among those who perceived handwashing to be effective and illness severity to be greater [77]. Similar observations were also indicated in a review of studies on handwashing practices in the community during and after the SARS outbreak, conducted by Fung and Cairncross [78], which stated that in eight studies, a significant gender-related difference was noted for compliance with handwashing and females were characterized by better behaviors.

It is stated that educational level may influence the hand hygiene practices of an individual. The study of Tao et al. [79] revealed that adults with a high level of education, especially those who had completed senior high school, college, or above, were more likely to wash their hands after defecation and before eating meals when compared to adults with a lower level of education. Similar results were observed in the study conducted among university students, as the level of education was found to be a significant predictor of handwashing practices and students with higher-grade education scored higher in handwashing practice assessment than those with lower-grade education [33]. However, an inverse relationship between the level of professional educational and the rate of handwashing compliance was shown in the study of Duggan et al. [80], as the authors found that nurses were characterized by significantly higher rate of handwashing compliance (91.3%) when compared to physicians (72.4%).

It may be stated that, in general, children and adolescents tend to have rather selective knowledge concerning proper hand hygiene behaviors. In the study of Lopez-Quintero et al. [81] conducted in a group of primary school students, most students reported handwashing after using the toilet (82.5%), while a minor share of respondents declared that they washed their hands with soap after leaving the bathroom (57.4%). Another study of Peltzer and Pengpid [82] confirmed that hand hygiene practices applied by adolescents are not satisfactory, as 59.8% of the study population did not always wash their hands with soap, 45.2% did not always wash their hands before meals, and 26.5% did not always wash their hands after using the toilet. However, in a study carried out among Turkish adolescents, adequate hand hygiene practices were observed, as a predominant share of respondents were aware of the exact time of handwashing required and declared that they washed their hands with water and soap [83].

In the present study, except for using a face mask, various protective measures were studied, including handwashing (most commonly declared), not leaving home, avoiding public places, avoiding contact with those who may be sick, using alcohol-based hand rub, using gloves, not touching the face, and taking medications or dietary supplements (less commonly declared).

Surprisingly, a similar frequency of response (about 30–40%) was observed for not touching the face and taking medications or dietary supplements. While not touching the face is indicated as an essential measure to reduce the transmission of infections [84], there are no proven effective therapeutic agents available for use to prevent COVID-19 infection [85].

The poor practicing of protective behaviors declared by adolescents in the present study may result from the fact that their knowledge about them is still insufficient; however, in our study, female respondents were generally characterized by a higher level of knowledge concerning hand hygiene and protective behaviors. It seems that the share of female respondents presenting sufficient level of knowledge was higher than the male respondents, as it was observed for the protective behaviors. Similar observations were indicated in a study conducted in the USA, as during the early days of the COVID-19 pandemic, the increased knowledge of participants was associated with decreased participation in purchasing more goods and in attending large gatherings [86]. In the mentioned study, among the respondents with higher level of knowledge, only a lower share used masks when compared to the proportion of respondents with a lower level of knowledge [86], but this reverse situation is explained by the authors as resulting from the lack of supply of masks combined with the recommendations of USA authorities for the general population to not use masks so that they are saved for frontline healthcare workers [87].

In the present study, which analyzed students’ knowledge and their beliefs regarding the choice of method that is better for proper hand hygiene and personal protection, a number of respondents reported that in their opinion, handwashing is as good as using gloves and using soap is as good as using alcohol-based hand rub. Fortunately, when asked about their actual behaviors, the number of adolescents who declared handwashing was higher than those who declared using gloves or using alcohol-based hand rub. However, this may have resulted from the supply shortage (as indicated in the USA study in the case of face masks [87]) in Poland, especially noticed for alcohol-based hand rub, which was rarely available and extremely expensive in the studied period [88]. Taking this into account, it may be supposed that handwashing was used as the main preventive measure not because it is the recommended behavior [3], but because it is the cheapest and easiest way to provide protection.

When asked about their handwashing behaviors, female respondents declared that they more often practiced the recommended behaviors than males, which is associated with the fact that independent of the country [89], gender is indicated as an important determinant [90]. Similarly, the level of their knowledge in this area was higher, which is in agreement with the results of other authors also indicating that women have a higher level of knowledge than men [28]. Therefore, it can be emphasized that education is crucial [91]—especially for men. Most importantly, during this COVID-19 pandemic, education should be provided not only to children but also to adolescents and adults [92].

Although the present study provided novel information about hand hygiene and personal protection in the population of adolescents during the COVID-19 pandemic, it has some limitations. First, the study was conducted only in a population of one country, so it provides detailed information only about that specific population and should be reproduced in other countries. Moreover, the study focused only on adolescents, while, in reality, hand hygiene and personal protection are even more important in other age groups, especially the elderly. Last but not least, the study assessed only declared behaviors that were not verified using direct observational research techniques.

However, some recommendations may be formulated based on the data obtained from the study. Irrespective of gender, some false beliefs and improper behaviors were observed among the participants, so proper education is crucial, especially during the COVID-19 global pandemic. Furthermore, compared to males, females were characterized by a higher level of knowledge and exhibited better hand hygiene and personal protective behaviors, so dedicated education should be provided mainly for secondary school males.

## 5. Conclusions

The study showed that female secondary school students were characterized by a higher level of knowledge on hand hygiene and personal protection, and they exhibited better behaviors when compared to male respondents. However, irrespective of gender, some false beliefs and improper behaviors were observed among the respondents; thus, education is necessary, especially in the period of the COVID-19 pandemic.

## Figures and Tables

**Table 1:** Sampling of Polish secondary schools included in the Polish Adolescents’ COVID-19 Experience (PLACE-19) Study for the assessment of hand hygiene and personal protective behaviors.

Level	1st Stage (31 March–14 April 2020)	2nd Stage (15 April–29 April 2020)
Voivodeships	All voivodeships (*n* = 16) included	Voivodeships with an inadequate number of completed questionnaires with no missing data (less than 50) after the 1st stage (*n* = 10) included
Counties	Random sampling of 5 counties for each of the 16 voivodeships (*n* = 80)	Random sampling of 5 counties for each of the 10 voivodeships (*n* = 50)
Secondary schools	Random sampling of 5 schools for each of the 80 counties (*n* = 400)	Random sampling of 5 schools for each of the 50 counties (*n* = 250)

**Table 2:** Knowledge and beliefs of the questioned sample of Polish secondary school students based on the choice of method which in their opinion is better for proper hand hygiene and personal protection (ensures better protection).

Choice of Method That Is Better for Proper Hand Hygiene and Personal Protection	Males (*n* = 814)	Females (*n* = 1509)	*p* **
Not leaving home *	542 (66.6%)	1052 (69.7%)	0.0521
Using a face mask	45 (5.5%)	50 (3.3%)	
Behaviors are equally good	199 (24.4%)	364 (24.1%)	
Refuse answering (do not know)	28 (3.4%)	43 (2.8%)	
Handwashing *	276 (33.9%)	392 (26.0%)	<0.0001
Using gloves	77 (9.5%)	108 (7.2%)	
Behaviors are equally good	428 (52.6%)	959 (63.6%)	
Refuse answering (do not know)	33 (4.1%)	50 (3.3%)	
Using soap *	181 (22.2%)	383 (25.4%)	<0.0001
Using alcohol-based hand rub	186 (22.9%)	217 (14.4%)	
Behaviors are equally good	399 (49.0%)	852 (56.5%)	
Refuse answering (do not know)	48 (5.9%)	57 (3.8%)	
Using liquid soap *	387 (47.5%)	776 (51.4%)	0.0001
Using a soap bar	79 (9.7%)	74 (4.9%)	
Behaviors are equally good	292 (35.9%)	529 (35.1%)	
Refuse answering (do not know)	56 (6.9%)	130 (8.6%)	
Using paper towels *	359 (44.1%)	756 (50.1%)	0.0441
Using a hand dryer	158 (19.4%)	254 (16.8%)	
Behaviors are equally good	194 (23.8%)	336 (22.3%)	
Refuse answering (do not know)	103 (12.7%)	163 (10.8%)	

* Answers interpreted as proper behavior; ** chi^2^ test.

**Table 3:** Knowledge and beliefs of the questioned sample of Polish secondary school students based on the time of handwashing which in their opinion is required.

Time of Handwashing	Males (*n* = 814)	Females (*n* = 1509)	*p* **
Less than 5 s	3 (0.4%)	5 (0.3%)	<0.0001
5–10 s	31 (3.8%)	29 (1.9%)	
11–20 s	97 (11.9%)	153 (10.1%)	
21–40 s *	536 (65.8%)	1120 (74.2%)	
More than 40 s *	82 (10.1%)	144 (9.5%)	
Time does not matter	37 (4.5%)	20 (1.3%)	
I don’t know	28 (3.4%)	38 (2.5%)	
Wrong answers	168 (20.6%)	207 (13.7%)	<0.0001
Proper answers	618 (75.9%)	1264 (83.8%)	
I don’t know	28 (3.4%)	38 (2.5%)	

* Answer interpreted as proper behavior (proper answers); ** chi^2^ test.

**Table 4:** Personal protective behaviors declared by the questioned sample of Polish secondary school students, before and after the implementation of the legal regulation that the nose and mouth should be covered in public places.

Responses by the Sample Questioned before the Implementation of the Legal Regulation *	**Males** **(*n* = 452)**	**Females** **(*n* = 862)**	*p* **
Not leaving home	387 (85.6%)	788 (91.4%)	0.0012
Using a face mask	116 (25.7%)	300 (34.8%)	0.0007
Not touching the face	170 (37.6%)	396 (45.9%)	0.0038
Using gloves	164 (36.3%)	399 (46.3%)	0.0005
Handwashing	428 (94.7%)	845 (98.0%)	0.0009
Using alcohol-based hand rub	278 (61.5%)	667 (77.4%)	<0.0001
Avoiding contact with those who may be sick	320 (70.8%)	677 (78.5%)	0.0018
Avoiding public places	367 (81.2%)	760 (88.2%)	0.0006
Taking medications or dietary supplements	142 (31.4%)	346 (40.1%)	0.0019
Other	3 (0.7%)	8 (0.9%)	0.6171
Responses by the Sample Questioned after the Implementation of the Legal Regulation *	**Males** **(*n* = 362)**	**Females** **(*n* = 647)**	p **
Not leaving home	229 (63.3%)	480 (74.2%)	0.0003
Using a face mask	310 (85.6%)	575 (88.9%)	0.1331
Not touching the face	135 (37.3%)	280 (43.3%)	0.0639
Using gloves	207 (57.2%)	433 (66.9%)	0.0021
Handwashing	339 (93.6%)	633 (97.8%)	0.0007
Using alcohol-based hand rub	241 (66.6%)	497 (76.8%)	0.0004
Avoiding contact with those who may be sick	241 (66.6%)	502 (77.6%)	0.0001
Avoiding public places	224 (61.9%)	488 (75.4%)	<0.0001
Taking medications or dietary supplements	81 (22.4%)	208 (32.1%)	0.0010
Other	2 (0.6%)	0 (0.0%)	0.0584

* Before/after 16 April 2020 [43]; ** chi^2^ test.

**Table 5:** Personal protective behaviors of vulnerable relatives declared by the questioned sample of Polish secondary school students, before and after the implementation of the legal regulation that the nose and mouth should be covered in public places.

Responses by Sample Questioned before the Implementation of the Legal Regulation *	**Males** **(*n* = 452)**	**Females** **(*n* = 862)**	*p* **
Not leaving home	350 (77.4%)	673 (78.1%)	0.7899
Using a face mask	210 (46.5%)	476 (55.2%)	0.0025
Not touching the face	211 (46.7%)	440 (51.0%)	0.1329
Using gloves	270 (59.7%)	632 (73.3%)	<0.0001
Handwashing	430 (95.1%)	835 (96.9%)	0.1149
Using alcohol-based hand rub	336 (74.3%)	705 (81.8%)	0.0016
Avoiding contact with those who may be sick	330 (73.0%)	690 (80.0%)	0.0004
Avoiding public places	322 (71.2%)	586 (68.0%)	0.0257
Taking medications or dietary supplements	184 (40.7%)	384 (44.5%)	0.0020
Other	1 (0.2%)	3 (0.3%)	0.8963
Responses by Sample Questioned after the Implementation of the Legal Regulation *	**Males** **(*n* = 362)**	**Females** **(*n* = 647)**	*p* **
Not leaving home	212 (58.6%)	426 (65.8%)	0.0214
Using a face mask	339 (93.6%)	613 (94.7%)	0.4683
Not touching the face	179 (49.4%)	313 (48.4%)	0.7447
Using gloves	276 (76.2%)	552 (85.3%)	0.0003
Handwashing	342 (94.5%)	628 (97.1%)	0.0408
Using alcohol-based hand rub	270 (74.6%)	513 (79.3%)	0.0857
Avoiding contact with those who may be sick	242 (66.9%)	481 (74.3%)	0.0113
Avoiding public places	198 (54.7%)	383 (59.2%)	0.1653
Taking medications or dietary supplements	103 (28.5%)	257 (39.7%)	0.0003
Other	2 (0.6%)	0 (0.0%)	0.0584

* Before/after 16 April 2020 [43]; ** chi^2^ test.

**Table 6:** Frequency of handwashing declared by the questioned sample of Polish secondary school students.

Declared Frequency	Males (*n* = 814)	Females (*n* = 1509)	*p* *
Not washing at all	0 (0.0%)	0 (0.0%)	<0.0001
1–2 times	26 (3.2%)	19 (1.3%)	
3–5 times	174 (21.4%)	196 (13.0%)	
6–10 times	308 (37.8%)	524 (34.7%)	
11–15 times	163 (20.0%)	362 (24.0%)	
16–20 times	68 (8.4%)	222 (14.7%)	
21–30 times	44 (5.4%)	119 (7.9%)	
More than 30 times	31 (3.8%)	67 (4.4%)	

* chi^2^ test.

**Table 7:** Reasons for not handwashing declared by the questioned sample of Polish secondary school students.

Declared Reasons	Males (*n* = 814)	Females (*n* = 1509)	*p* ***
In my opinion there is no need to do it	113 (13.9%)	87 (5.8%)	<0.0001
I don’t feel like doing it	67 (8.2%)	54 (3.6%)	<0.0001
I have no time to do it	37 (4.5%)	21 (1.4%)	<0.0001
I am forgetting about it	251 (30.8%)	334 (22.1%)	<0.0001
It is constricted	21 (2.6%)	44 (2.9%)	0.6398
Due to side effects	42 (5.2%)	114 (7.6%)	0.0278
Other *	11 (1.4%)	18 (1.2%)	0.7424
Various reasons for not washing **	374 (45.9%)	480 (31.8%)	<0.0001
I always wash my hands	440 (54.1%)	1029 (68.2%)	

* Various non-interpretable answers; ** number of respondents combined for various reasons declared; *** chi^2^ test.

**Table 8:** Circumstances of handwashing associated with socializing and being exposed to contact with other people, as declared by the questioned sample of Polish secondary school students, based on the Handwashing Habits Questionnaire [56].

Characteristics		Males (*n* = 814)	Females (*n* = 1509)	*p* *
After coming back home	Never	10 (1.2%)	6 (0.4%)	<0.0001
	Sometimes	113 (13.9%)	101 (6.7%)	
	Always	691 (84.9%)	1402 (92.9%)	
After handshaking	Never	152 (18.7%)	212 (14.0%)	0.0041
	Sometimes	330 (40.5%)	598 (39.6%)	
	Always	332 (40.8%)	699 (46.3%)	
After using public transportation	Never	67 (8.2%)	57 (3.8%)	<0.0001
	Sometimes	164 (20.1%)	173 (11.5%)	
	Always	583 (71.6%)	1279 (84.8%)	
After money exchange	Never	186 (22.9%)	189 (12.5%)	<0.0001
	Sometimes	257 (31.6%)	421 (27.9%)	
	Always	371 (45.6%)	899 (59.6%)	

* chi^2^ test.

**Table 9:** Circumstances of handwashing associated with health, as declared by the questioned sample of Polish secondary school students, based on the Handwashing Habits Questionnaire [56].

Characteristics		Males (*n* = 814)	Females (*n* = 1509)	*p* *
Before touching sick people	Never	222 (27.3%)	356 (23.6%)	0.1156
	Sometime	228 (28.0%)	464 (30.7%)	
	Always	364 (44.7%)	689 (45.7%)	
After touching sick people	Never	43 (5.3%)	49 (3.2%)	0.0168
	Sometimes	100 (12.3%)	157 (10.4%)	
	Always	671 (82.4%)	1303 (86.3%)	
After nose blowing	Never	168 (20.6%)	244 (16.2%)	0.0004
	Sometimes	369 (45.3%)	634 (42.0%)	
	Always	277 (34.0%)	631 (41.8%)	
After sneezing	Never	145 (17.8%)	203 (13.5%)	0.0006
	Sometimes	348 (42.8%)	597 (39.5%)	
	Always	321 (39.4%)	709 (47.0%)	
After coughing	Never	162 (19.9%)	241 (16.0%)	0.0069
	Sometimes	354 (43.5%)	625 (41.4%)	
	Always	298 (36.6%)	643 (42.6%)	

* chi^2^ test.

**Table 10:** Procedure of handwashing declared by the questioned sample of Polish secondary school students.

Characteristics		Males (*n* = 814)	Females (*n* = 1509)	*p* **
Folding sleeves	Never	37 (4.5%)	39 (2.6%)	0.0045
	Sometimes	166 (20.4%)	314 (20.8%)	
	Always	388 (47.7%)	855 (56.7%)	
	Not applicable	223 (27.4%)	301 (19.9%)	
Removing watches and bracelets	Never	128 (8.5%)	81 (10.0%)	0.0053
	Sometimes	327 (21.7%)	128 (15.7%)	
	Always	199 (13.2%)	118 (14.5%)	
	Not applicable	855 (56.7%)	487 (59.8%)	
Removing rings before or during handwashing	Never	29 (3.6%)	190 (12.6%)	0.0079
	Sometimes	34 (4.2%)	132 (8.7%)	
	Always	64 (7.9%)	197 (13.1%)	
	Not applicable	687 (84.4%)	990 (65.6%)	
Using soap	Never	5 (0.6%)	1 (0.1%)	0.0185
	Sometimes	48 (5.9%)	70 (4.6%)	
	Always	761 (93.5%)	1438 (95.3%)	
Using warm water	Never	28 (3.4%)	15 (1.0%)	0.0001
	Sometimes	279 (34.3%)	498 (33.0%)	
	Always	507 (62.3%)	996 (66.0%)	
Soaking hands before using soap	Never	88 (10.8%)	113 (7.5%)	0.0064
	Sometimes	156 (19.2%)	259 (17.2%)	
	Always	568 (69.8%)	1136 (75.3%)	
	Not applicable	2 (0.2%)	1 (0.1%)	
Spreading soap lather throughout the hands	Never	47 (5.8%)	52 (3.4%)	0.0163
	Sometimes	286 (35.1%)	510 (33.8%)	
	Always	480 (59.0%)	947 (62.8%)	
	Not applicable	1 (0.1%)	0 (0.0%)	
Turning the faucet off with hands *	Never	142 (17.4%)	363 (24.1%)	0.0004
	Sometimes	219 (26.9%)	409 (27.1%)	
	Always	453 (55.7%)	737 (48.8%)	
Drying hands with a towel	Never	13 (1.6%)	44 (2.9%)	0.0120
	Sometimes	114 (14.0%)	262 (17.4%)	
	Always	687 (84.4%)	1203 (79.7%)	

* Including in the applied procedure was interpreted as incorrect, as the faucet should not be touched barehanded after handwashing; ** chi^2^ test.

## Supplementary Materials

The following are available online at https://www.mdpi.com/1660-4601/17/16/5770/s1. Table S1. The questions on hand hygiene and personal protective behaviors knowledge and beliefs that were asked. Table S2. The questions on actual hand hygiene and personal protective behaviors that were asked. Table S3. Frequency of handwashing declared by the questioned sample of Polish secondary school students for the period before the COVID-19 pandemic and during the COVID-19 global pandemic. Table S4. Reasons for not handwashing declared by the questioned sample of Polish secondary school students for the period before the COVID-19 pandemic and during the COVID-19 global pandemic. Table S5. Circumstances of handwashing associated with socializing and being exposed to contact with other people, as declared by the questioned sample of Polish secondary school students, based on the Handwashing Habits Questionnaire [56] for the period before the COVID-19 pandemic and during the COVID-19 global pandemic. Table S6. Circumstances of handwashing associated with health, as declared by the questioned sample of Polish secondary school students, based on the Handwashing Habits Questionnaire [56] for the period before the COVID-19 pandemic and during the COVID-19 global pandemic. Table S7. Procedure of handwashing declared by the questioned sample of Polish secondary school students for the period before the COVID-19 pandemic and during the COVID-19 global pandemic.
