# Supplementary material for: Analysis of Gender-Dependent Personal Protective Behaviors in a National Sample: Polish Adolescents’ COVID-19 Experience (PLACE-19) Study
Source: Int J Environ Res Public Health. 2020 Aug 10;17(16):5770. doi: 10.3390/ijerph17165770 (PMC7459707; doi:10.3390/ijerph17165770)
Supplement: Supplementary file 1 [file ijerph-17-05770-s001.pdf]

International Journal of
Environmental Research and Public Health

Article

Analysis of Gender-Dependent Personal Protective Behaviors in a National Sample: Polish Adolescents' COVID-19 Experience (PLACE-19) Study

Supplementary Table 1. The questions on hand hygiene and personal protective behaviors knowledge and beliefs that were asked.

Question		Answers to be chosen
Choice of method which in their opinion is better for proper hand hygiene and personal protection (ensuring better protection)	Not leaving home vs. using face mask (close-ended question)	□ Not leaving home<br>□ Using face mask<br>□ They are equally good<br>□ Do not know which one is better
	Handwashing vs. using gloves (close-ended question)	□ Handwashing<br>□ Using gloves<br>□ They are equally good<br>□ Do not know
	Using soap vs. using alcohol-based hand rub (close-ended question)	□ Using soap<br>□ Using alcohol-based hand rub<br>□ They are equally good<br>□ Do not know
	Using liquid soap vs. using soap bar (close-ended question)	□ Using liquid soap<br>□ Using soap bar<br>□ They are equally good<br>□ Do not know
	Using paper towel vs. using hand dryer (close-ended question)	□ Using paper towel<br>□ Using hand dryer<br>□ They are equally good<br>□ Do not know
Required time of handwashing in their opinion (close-ended question)		□ Less than 5 seconds<br>□ 5–10 seconds<br>□ 11–20 seconds<br>□ 21–40 seconds<br>□ More than 40 seconds<br>□ Time does not matter<br>□ Do not know

Supplementary Table 2. The questions on actual hand hygiene and personal protective behaviors that were asked.

Question	Answers to be chosen	
Personal protective behaviors (multiple-choice question)*	□ Not leaving home<br>□ Using face mask<br>□ Not touching face<br>□ Using gloves<br>□ Handwashing<br>□ Using alcohol-based hand rub<br>□ Avoiding contact with those who may be sick<br>□ Avoiding public places<br>□ Taking medications or dietary supplements<br>□ Other	
Frequency of washing hands (close-ended question) **	□ Not washing at all<br>□ 1–2 times a day<br>□ 3–5 times a day<br>□ 6–10 times a day<br>□ 11–15 times a day<br>□ 16–20 times a day<br>□ 21–30 times a day<br>□ More than 30 times a day	
Reasons for not washing hands (multiple-choice question) **	□ In my opinion, there is no need to do it<br>□ I don't feel like doing it<br>□ I have no time to do it<br>□ I am forgetting about it<br>□ It is constricted (e.g., there is no soap, there is no bathroom nearby)<br>□ Due to side effects (e.g., skin problems due to frequent contact with soap)<br>□ Other<br>□ I always wash my hands	
Circumstances of washing hands **	After coming back home	□ Never<br>□ Sometimes<br>□ Always
	After handshaking	□ Never<br>□ Sometimes<br>□ Always
	After using public transportation	□ Never<br>□ Sometimes<br>□ Always
	After money exchange	□ Never<br>□ Sometimes<br>□ Always
	Before touching sick people	□ Never<br>□ Sometimes<br>□ Always
	After touching sick people	□ Never<br>□ Sometimes<br>□ Always
	After blowing nose	□ Never<br>□ Sometimes

Question	Answers to be chosen	
		□ Always
	After sneezing	□ Never<br>□ Sometimes<br>□ Always
	After coughing	□ Never<br>□ Sometimes<br>□ Always
Procedure applied for washing hands **	Folding sleeves	□ Never<br>□ Sometimes<br>□ Always<br>□ Not applicable
	Removing watch and bracelets	□ Never<br>□ Sometimes<br>□ Always<br>□ Not applicable
	Removing rings before or during handwashing	□ Never<br>□ Sometimes<br>□ Always<br>□ Not applicable
	Using soap	□ Never<br>□ Sometimes<br>□ Always
	Using warm water	□ Never<br>□ Sometimes<br>□ Always
	Soaking hands before using soap	□ Never<br>□ Sometimes<br>□ Always<br>□ Not applicable (I do not use soap)
	Spreading soap lather throughout the hands	□ Never<br>□ Sometimes<br>□ Always<br>□ Not applicable (I do not use soap)
	Turning the faucet off with hand	□ Never<br>□ Sometimes<br>□ Always
	Drying hands with towel	□ Never<br>□ Sometimes<br>□ Always

* respondents were asked about them and their vulnerable relatives (e.g., elderly ones in their families);.** respondents were asked about period during the COVID-19 pandemic and before the COVID-19 pandemic.

Supplementary Table 3. Frequency of washing hands declared by the questioned sample of Polish secondary school students for the period before the COVID-19 issue and during the COVID-19 global pandemic.

Declared frequency	Boys (n = 814)		p *	Girls (n = 1,509)		p *
	Before the COVID-19	During the COVID-19		Before the COVID-19	During the COVID-19	
Not washing at all	5 (0.6%)	0 (0.0%)	< 0.0001	4 (0.3%)	0 (0.0%)	< 0.0001
1–2 times	135 (16.6%)	26 (3.2%)		130 (8.6%)	19 (1.3%)	
3–5 times	345 (42.4%)	174 (21.4%)		593 (39.3%)	196 (13.0%)	
6–10 times	203 (24.9%)	308 (37.8%)		435 (28.8%)	524 (34.7%)	
11–15 times	76 (9.3%)	163 (20.0%)		213 (14.1%)	362 (24.0%)	
16–20 times	25 (3.1%)	68 (8.4%)		84 (5.6%)	222 (14.7%)	
21–30 times	11 (1.4%)	44 (5.4%)		30 (2.0%)	119 (7.9%)	
> 30 times	14 (1.7%)	31 (3.8%)		20 (1.3%)	67 (4.4%)	

* chi^2 test.

Supplementary Table 4. Reasons for not washing hands declared by the questioned sample of Polish secondary school students for the period before the COVID-19 issue and during the COVID-19 global pandemic.

**Declared reasons**	**Boys (n = 814)**		*p* ***	**Girls (n = 1,509)**		*p* ***
	**Before the COVID-19**	**During the COVID-19**		**Before the COVID-19**	**During the COVID-19**	
In my opinion there is no need to do it	159 (19.5%)	113 (13.9%)	0.0001	178 (11.8%)	87 (5.8%)	< 0.0001
I don't feel like doing it	132 (16.2%)	67 (8.2%)		166 (11.0%)	54 (3.6%)	
I have no time to do it	80 (9.8%)	37 (4.5%)		86 (5.7%)	21 (1.4%)	
I am forgetting about it	281 (34.5%)	251 (30.8%)		480 (31.8%)	334 (22.1%)	
It is constricted	36 (4.4%)	21 (2.6%)		112 (7.4%)	44 (2.9%)	
Due to side effects	36 (4.4%)	42 (5.2%)		112 (7.4%)	114 (7.6%)	
Other *	3 (0.4%)	11 (1.4%)		12 (0.8)	18 (1.2%)	
Various reasons for not washing **	484 (40.5%)	374 (45.9%)	< 0.0001	802 (53.1%)	480 (31.8%)	< 0.0001
I always wash my hands	330 (40.5%)	440 (54.1%)		707 (46.9%)	1,029 (68.2%)	

* various not interpretable answers; ** number of respondents combined for various reasons declared; *** chi^2 test.

Supplementary Table 5. Circumstances of washing hands, associated with socializing and being exposed to contact with other people, declared by the questioned sample of Polish secondary school students, based on Handwashing Habits Questionnaire [56] for the period before the COVID-19 issue and during the COVID-19 global pandemic.

Characteristics	Boys (n = 814)		*p* *	Girls (n = 1,509)		*p* *
	Before the COVID-19	During the COVID-19		Before the COVID-19	During the COVID-19	
**After coming back home**						
Never	48 (7.5%)	10 (1.2%)		38 (2.5%)	6 (0.4%)	
Sometimes	260 (31.0%)	113 (13.9%)	< 0.0001	429 (28.4%)	101 (6.7%)	< 0.0001
Always	506 (61.5%)	691 (84.9%)		1.042 (69.1%)	1.402 (92.9%)	
**After handshaking**						
Never	301 (37.0%)	152 (18.7%)		565 (37.4%)	212 (14.0%)	
Sometimes	366 (45.0%)	330 (40.5%)	< 0.0001	657 (43.5%)	598 (39.6%)	< 0.0001
Always	147 (18.1%)	332 (40.8%)		287 (19.0%)	699 (46.3%)	
**After using public transportation**						
Never	136 (16.7%)	67 (8.2%)		174 (11.5%)	57 (3.8%)	
Sometimes	336 (41.3%)	164 (20.1%)	< 0.0001	561 (37.2%)	173 (11.5%)	< 0.0001
Always	342 (42.0%)	583 (71.6%)		774 (51.3%)	1,279 (84.8%)	
**After money exchange**						
Never	311 (38.2%)	186 (22.9%)		484 (32.1%)	189 (12.5%)	
Sometimes	310 (38.1%)	257 (31.6%)	< 0.0001	566 (37.5%)	421 (27.9%)	< 0.0001
Always	193 (23.7%)	371 (45.6%)		459 (30.4%)	899 (59.6%)	

* chi^2 test.

Supplementary Table 6. Circumstances of washing hands, associated with health, declared by the questioned sample of Polish secondary school students, based on Handwashing Habits Questionnaire [56] for the period before the COVID-19 issue and during the COVID-19 global pandemic.

Characteristics	Boys (n = 814)		*p* *	Girls (n = 1,509)		*p* *
	Before the COVID-19	During the COVID-19		Before the COVID-19	During the COVID-19	
**Before touching sick people**						
Never	268 (32.9%)	222 (27.3%)		528 (35.0%)	356 (23.6%)	
Sometimes	288 (35.4%)	228 (28.0%)	< 0.0001	505 (33.5%)	464 (30.7%)	< 0.0001
Always	258 (31.7%)	364 (44.7%)		476 (31.5%)	689 (45.7%)	
**After touching sick people**						
Never	76 (9.3%)	43 (5.3%)		141 (9.3%)	49 (3.2%)	
Sometimes	228 (28.0%)	100 (12.3%)	< 0.0001	403 (26.7%)	157 (10.4%)	< 0.0001
Always	510 (62.7%)	671 (82.4%)		965 (63.9%)	1,303 (86.3%)	
**After blowing nose**						
Never	246 (30.2%)	168 (20.6%)		416 (27.6%)	244 (16.2%)	
Sometimes	364 (44.7%)	369 (45.3%)	< 0.0001	664 (44.0%)	634 (42.0%)	< 0.0001
Always	204 (25.1%)	277 (34.0%)		429 (28.4%)	631 (41.8%)	
**After sneezing**						
Never	200 (24.6%)	145 (17.8%)		394 (26.1%)	203 (13.5%)	
Sometimes	391 (48.0%)	348 (42.8%)	< 0.0001	657 (43.5%)	597 (39.5%)	< 0.0001
Always	223 (27.4%)	321 (39.4%)		458 (30.4%)	709 (47.0%)	
**After coughing**						
Never	235 (28.9%)	162 (19.9%)		458 (30.4%)	241 (16.0%)	
Sometimes	376 (46.2%)	354 (43.5%)	< 0.0001	633 (41.9%)	625 (41.4%)	< 0.0001
Always	203 (24.9%)	298 (36.6%)		418 (27.7%)	643 (42.6%)	

* chi^2 test.

Supplementary Table 7. Procedure of handwashing declared by the questioned sample of Polish secondary school students for the period before the COVID-19 issue and during the COVID-19 global pandemic.

Characteristics	Boys (n = 814)		p ***	Girls (n = 1,509)		p ***
	Before the COVID-19	During the COVID-19		Before the COVID-19	During the COVID-19	
**Folding sleeves**						
Never	62 (7.6%)	37 (4.5%)	0.0095	92 (6.1%)	39 (2.6%)	< 0.0001
Sometimes	194 (23.8%)	166 (20.4%)		403 (26.7%)	314 (20.8%)	
Always	366 (45.0%)	388 (47.7%)		799 (52.9%)	855 (56.7%)	
Not applicable	192 (23.6%)	223 (27.4%)		215 (14,2%)	301 (19.9%)	
**Removing watch and bracelets**						
Never	87 (10.7%)	81 (10.0%)	0.6733	208 (13.8%)	190 (12.6%)	< 0.0001
Sometimes	134 (16.5%)	128 (15.7%)		316 (20.9%)	132 (8.7%)	
Always	130 (16.0%)	118 (14.5%)		191 (12.5%)	197 (13.1%)	
Not applicable	463 (56.9%)	487 (59.8%)		794 (52.6%)	990 (65.6%)	
**Removing rings before or during handwashing**						
Never	31 (3.8%)	29 (3.6%)	0.2346	229 (15.2%)	190 (12.6%)	0.0411
Sometimes	45 (5.5%)	34 (4.2%)		157 (10.4%)	132 (8.7%)	
Always	81 (10.0%)	64 (7.9%)		201 (13.3%)	197 (13.1%)	
Not applicable	657 (80.7%)	687 (84.4%)		922 (61.1%)	990 (65.6%)	
**Using soap**						
Never	10 (1.2%)	5 (0.6%)	< 0.0001	7 (0.5%)	1 (0.1%)	< 0.0001
Sometimes	134 (16.5%)	48 (5.9%)		195 (12.9%)	70 (4.6%)	
Always	670 (82.3%)	761 (93.5%)		1,307 (86.6%)	1,438 (95.3%)	
**Using warm water**						
Never	33 (4.1%)	28 (3.4%)	0.1276	28 (1.9%)	15 (1.0%)	0.0001
Sometimes	314 (38.6%)	279 (34.3%)		596 (39.5%)	498 (33.0%)	
Always	467 (57.4%)	507 (62.3%)		885 (58.6%)	996 (66.0%)	
**Soaking hands before using soap**						
Never	79 (9.7%)	88 (10.8%)	0.0205	119 (7.9%)	113 (7.5%)	0.0030
Sometimes	204 (25.1%)	156 (19.2%)		338 (22.4%)	259 (17.2%)	
Always	526 (64.6%)	568 (69.8%)		1,051 (69.6%)	1,136 (75.3%)	
Not applicable	5 (0.6%)	2 (0.2%)		1 (0.1%)	1 (0.1%)	
**Spreading soap lather throughout the hands**						
Never	74 (9.1%)	47 (5.8%)	< 0.0001	142 (9.4%)	52 (3.4%)	< 0.0001
Sometimes	341 (41.9%)	286 (35.1%)		659 (43.7%)	510 (33.8%)	
Always	394 (48.4%)	480 (59.0%)		704 (46.7%)	947 (62.8%)	
Not applicable	5 (0.6%)	1 (0.1%)		4 (0.3%)	0 (0.0%)	
**Turning the faucet off with hand** *						
Never	89 (10.9%)	142 (17.4%)	0.0001	210 (13.9%)	363 (24.1%)	< 0.0001
Sometimes	198 (24.3%)	219 (26.9%)		388 (25.7%)	409 (27.1%)	
Always	527 (64.7%)	453 (55.7%)		911 (60.4%)	737 (48.8%)	
**Drying hands with towel**						
Never	21 (2.6%)	13 (1.6%)	0.0008	56 (3.7%)	44 (2.9%)	0.0012
Sometimes	166 (20.4%)	114 (14.0%)		335 (22,2%)	262 (17.4%)	
Always	627 (77.0%)	687 (84.4%)		1,118 (74,1%)	1,203 (79.7%)	

* including in the applied procedure was interpreted as incorrect, as the faucet should not be touched barehanded after washing hands; ** chi^2 test.
